# Structured and Systematic Team and Procedure Training in Severe Trauma: Going from ‘Zero to Hero’ for a Time-Critical, Low-Volume Emergency Procedure Over Three Time Periods

**DOI:** 10.1007/s00268-021-05980-1

**Published:** 2021-02-10

**Authors:** Maryam Meshkinfamfard, Jon Kristian Narvestad, Johannes Wiik Larsen, Arezo Kanani, Jørgen Vennesland, Andreas Reite, Morten Vetrhus, Kenneth Thorsen, Kjetil Søreide

**Affiliations:** 1 Department of Gastrointestinal Surgery, Stavanger University Hospital, P.O. Box 8100, 4068 Stavanger, Norway; 2 Section for Traumatology, Surgical Clinic, Stavanger University Hospital, Stavanger, Norway; 3 Department of Surgery, Vascular & Thoracic Surgery Unit, Stavanger University Hospital, Stavanger, Norway; 4 Department of Clinical Science, University of Bergen, Bergen, Norway; 5 Department of Clinical Medicine, University of Bergen, Bergen, Norway

## Abstract

**Background:**

Resuscitative emergency thoracotomy is a potential life-saving procedure but is rarely performed outside of busy trauma centers. Yet the intervention cannot be deferred nor centralized for critically injured patients presenting in extremis. Low-volume experience may be mitigated by structured training. The aim of this study was to describe concurrent development of training and simulation in a trauma system and associated effect on one time-critical emergency procedure on patient outcome.

**Methods:**

An observational cohort study split into 3 arbitrary time-phases of trauma system development referred to as ‘early’, ‘developing’ and ‘mature’ time-periods. Core characteristics of the system is described for each phase and concurrent outcomes for all consecutive emergency thoracotomies described with focus on patient characteristics and outcome analyzed for trends in time.

**Results:**

Over the study period, a total of 36 emergency thoracotomies were performed, of which 5 survived (13.9%). The “early” phase had no survivors (0/10), with 2 of 13 (15%) and 3 of 13 (23%) surviving in the development and mature phase, respectively. A decline in ‘elderly’ (>55 years) patients who had emergency thoracotomy occurred with each time period (from 50%, 31% to 7.7%, respectively). The gender distribution and the injury severity scores on admission remained unchanged, while the rate of patients with signs on life (SOL) increased over time.

**Conclusion:**

The improvement over time in survival for one time-critical emergency procedure may be attributed to structured implementation of team and procedure training. The findings may be transferred to other low-volume regions for improved trauma care.

**Supplementary Information:**

The online version contains supplementary material available at (doi:10.1007/s00268-021-05980-1).

## Introduction

Trauma is a major health burden worldwide [1]. Severe injuries may cause sudden change to vital functions with risk of imminent death if no intervention or appropriate resuscitation takes place [2]. Certain emergency procedures are considered lifesaving if and when performed for the right indications and with appropriate training [3]. Resuscitative emergency thoracotomy is one such procedure indicated for severely injured patients in extremis, but with variation in reported outcomes and its use still being debated [4–6]. Notably, outcomes are demonstrated to be better in large, urban, busy trauma centers, often with a high rate of penetrating injuries [6].

Historically, survival after resuscitative thoracotomy for blunt trauma has been very low (e.g. 1–2%) and deemed futile when no sign of life (SOL) on admission [7]. However, some centers reported higher survival rates after blunt trauma (12%) [8], with a collective review suggesting outcomes may be less dismal [4]. More recently, the German Trauma Registry reported a survival rate of 4.8% after blunt trauma, and 7.6% survival rate was reported in a US nationwide study based on administrative data [9]. However, the price of the occasional ‘miracle [10]’ continues to be debated [11, 12]. Also, indications and compliance to guidelines influence the outcome rates reported [13]. Quite clearly, deciding *not* to perform a resuscitative emergency thoracotomy in a situation where this would be (even only potentially in theory) lifesaving, is associated with an obvious 100% mortality [13].

Notably, performing a resuscitative emergency thoracotomy as a life-saving procedure is an urgent decision—the dying patient cannot be transferred nor deferred for later intervention. Accordingly, almost half of all emergency thoracotomies in the German trauma registry are reportedly done outside the supra-regional trauma centers [14], with about 11% being done in a local hospital. Data from a busy region in the United States covering 28 hospitals found that 10 centers did on average <2 such procedures per year—3 of these centers were designated as level I [14]. Hence, this procedure is—more often than not—done at low-frequency and with little regularity in real-life practice for many surgeons. To mitigate this, educational strategies and deliberate training would be necessary to optimize performance and enhance the chance for a favorable outcome. Being prepared is of the essence to provide timely care in an emergency.

The aim of this study is to describe the systematic changes to education and training over time in a maturing trauma system and its association with indications and outcomes in one time-critical and potentially life-saving yet rare emergency procedure for critically ill trauma patients.

## Methods

The study is based on the clinical evaluation of two previously reported consecutive cohorts [15, 16]. These two cohorts describe the patient characteristics and outcomes for resuscitative emergency thoracotomy. In the current study, we describe the concurrent institutional and structural changes to the trauma system with particular focus on the education and certification of health care providers in our system over a long time period.

### Ethics and study design

The study is a quality assurance project, hence not subject to formal review for acceptance by the Regional Ethics Committee (REK Helse Vest). The project was approved by the Institutional Data Protection Officer (Personvernombudet, SUS) at Stavanger University Hospital (SUH), as required by institutional protocol. As an observational study, the STROBE guidelines were consulted and applied, where applicable [17].

### Study hospital and population

Stavanger University Hospital is one of the largest (in patient volume) trauma hospitals in Norway and has a primary catchment area of about 375.000 inhabitants as sole health care provider, but receives patients from several counties and hospitals beyond the primary catchment area (about 500–600 K) due to availability of prehospital care services (one air-ambulance helicopter and one search-and-rescue helicopter is located in Stavanger), neurosurgical and neurointensive care capacity and surgical intensive care resources and a busy interventional radiology service in addition to 24/7/365 surgical services covering trauma care. The epidemiology of the trauma deaths and the injured population in our region has been described in detail previously [18–21], as has the trauma team activation (TTA) criteria and the use of a 2-tiered TTA approach [22]. SUH has had a local trauma registry in place since 2004 [22] and an additional fracture registry since 2006 [23]. Briefly, the registry includes several standard metrics of injured patients, such as injury severity score (ISS), New ISS (NISS), Revised trauma score (RTS) [24] and probability of survival (Ps) calculations per TRISS methodology [25, 26], as previously described [22].

### Time interval and incremental periods

The study includes all emergency thoracotomies from January 1st, 2000 to December 31st, 2018, as described in detail in two previous studies [15, 16]. For sake of the current investigation, the study period is split into 3 time-phases as the ‘early’ (2001–2005), the ‘developing’ (2006–2011) and the ‘mature’ (2012–2018) period. This distinction is arbitrary yet represents time spans during which several changes occurred. The first time period (“early phase”) coincide with the initial report [15] during which no formal trauma system existed nationally, regionally and was based on local enthusiasts. The subsequent time periods defined as ‘developing’ and ‘mature’ coincides with the second report [16] on emergency thoracotomy in trauma patients. The ‘developing’ phase was chosen for its initial formal attempt at shaping the trauma system, both nationally and regionally. The latter and ‘mature’ phase represents the period during which many of not most structures were implemented (See Table 1 for details).

**Table 1:** Implementation of certification and training over 3 temporal phases

Phase	Early	Developing	Mature
Incremental time-period	*2001–2005*	*2006–2011*	*2012–2018*
ATLS™ certification	*some*	*most*	*all*
ATLS instructors and courses	*none/few*	*some*	*Several* + *local courses*
DSTC^a^ participation	*rare*	*some/most*	*all*
Trauma team activation	*1-tier*	*1-tier → 2-tier*	*2-tier*
Team simulation training	*none*	*occasional*	*weekly*
Cadaver procedure training	*none*	*occasional*	*regular/weekly*
Trauma audits	Ad hoc	*regular*	*regular*
SUH Trauma Registry	*2004→*	→	→
*Concomitant national changes to trauma system*			
National trauma plan	*none/*ad hoc	*v.1 (2006)*	*v.2 (2016)*
Nat’l advisory unit on trauma			*Est. 2013→*
National Trauma Registry	*none*	*None*	*Est. 2014 →*

^a^Either as DSTC courses or similar (e.g. practice of hemostatci emergency surgery on a live pig model; course on warsurgery)

### Systems change over time with increased focus on education, training and simulation

Trauma surgery is not a designated specialty in Norway [27], nor is “acute care surgery”. Hence, general surgeons and designated subspecialties (typically gastrointestinal surgeons; vascular surgeons) are the ones responsible for trauma care [28].

During the study period, an increased effort was placed on certification of all surgical trainees in ATLS-principles [29], as well as the faculty/staff surgeons (Table 1). The specific contributions to certification, training and system evolution are presented, giving an overview of critical elements in developing both individual skills and team-training for a general overview, rather than an exhaustive list. Several animal training courses were held and attended over the period (live pig model) as part of the mandatory “War Surgery” course during surgical training, and later also as part of Definitive Surgical Trauma Course (DSTC™) or similarly structured trauma training (porcine model for hemostatic emergency procedures in trauma [30, 31]) with a complete local operating team attending to enhance team training and dynamics [30, 31]. Other local or regional courses (e.g. skills training in laparoscopic abdominal surgery) were exploited for double purposes, e.g. the animals where used to practice emergency procedures as an integrated part of the training after the laparoscopy training was completed.

Cadaver training at the Department of Pathology commenced in the early phase in an ad hoc manner and, subsequently, was structured and systematically implemented during the development phase. Initially, emergency thoracotomy, thoracic drains, abdominal and pelvic packing was practiced, and other procedures were added to the spectrum during the later phase.

Simulation in teams through local adaption of the BEST team-training concept (*BE*tter and *S*ystematic *T*rauma training [32, 33]) was initiated and eventually has become a regular, weekly event in the hospital [34]. This focuses primarily on team interaction, situational awareness, communication (such as; clear messages; closed loop communication etc.) and the non-technical skills of trauma management using live mannequins and cases from the local registry for simulation.

### Statistical analysis

Statistical analyses were performed by IBM SPSS for Mac v. 26. (Statistical Package for Social Sciences; Armonk, NY, USA: IBM Corp). Descriptive data were analyzed using on-parametric tests, using Kruskal–Wallis for analyses of continuous data across the 3 time-periods, or Chi-square for trend (2 degrees of freedom) for categorial variables. All tests were two-tailed, and statistical significance attributed to *P* <0.050.

## Results

**Fig. 1:**
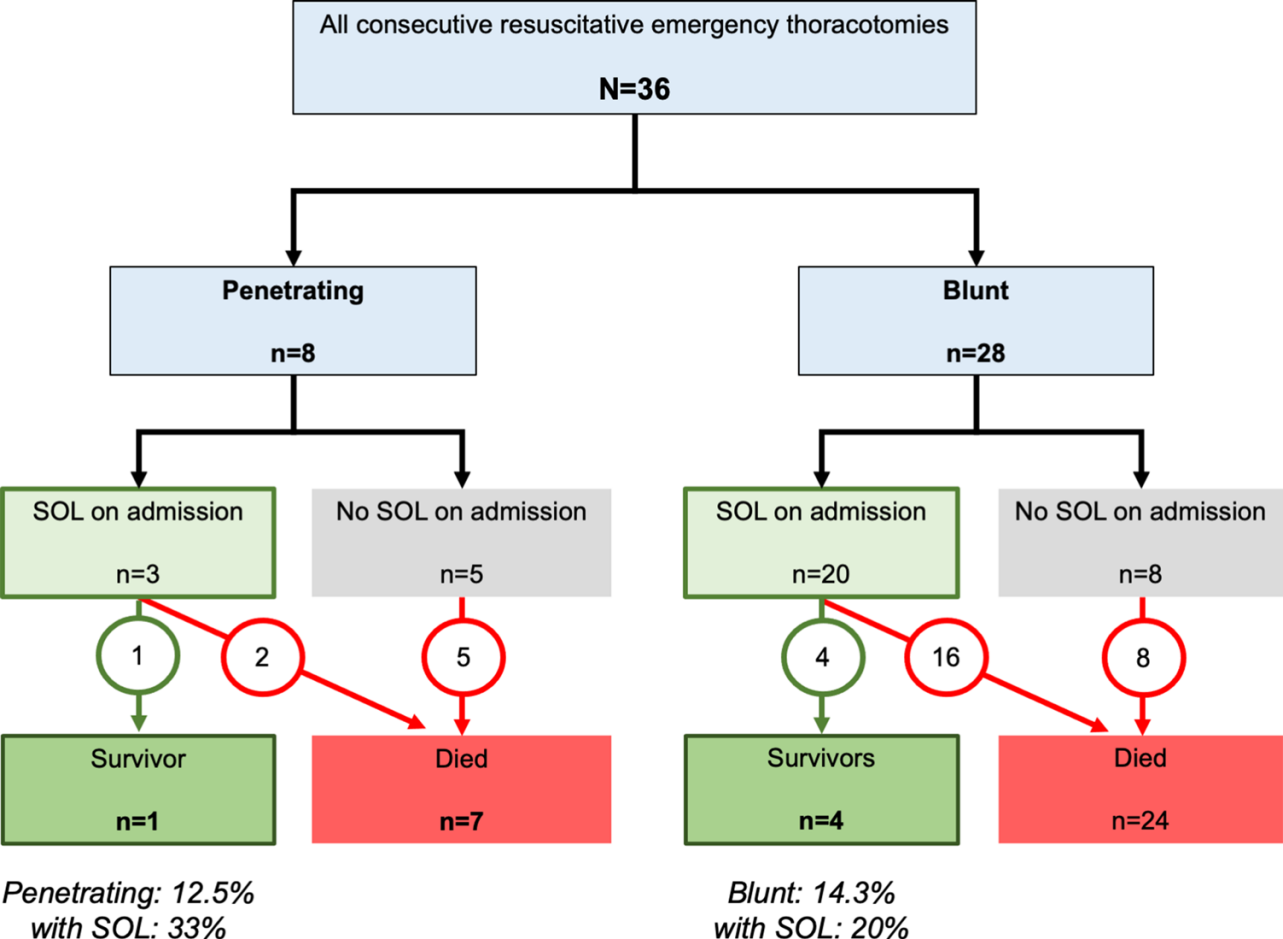
Flowchart of all patients included in the cohort *Legend* SOL denotes Signs of Life on admission

**Fig. 2:**
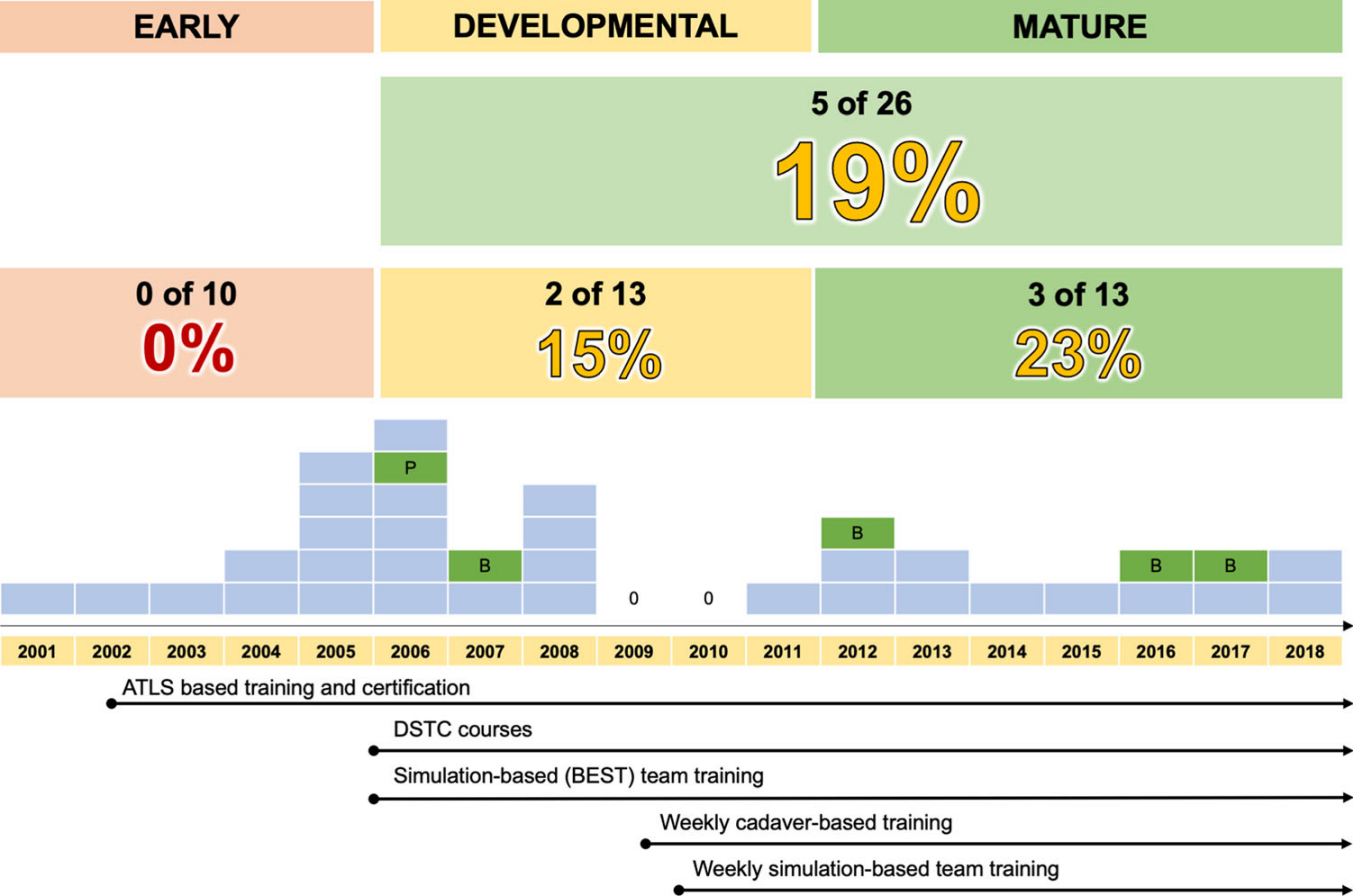
Time-dependent phases of change and associated outcomes *Legend* DSTC denotes ‘damage surgical trauma course’ or similar courses on hemostatic trauma surgery procedures on a live porcine model; ATLS denotes advanced Trauma Life Support; BEST denotes Better and Systematic Trauma training. Please see maintext for details

During the study period, there were a total of 36 injured patients who had an emergency thoracotomy (Fig. 1). The year-on-year number of procedures and associated system changes are presented in Fig. 2. On average, 2 procedures per year were performed. For the complete study period, the median age was 40 years (iqr 24–57), 27 were men (75%). Injury severity score (ISS) was median 40 (iqr 30–57), and NISS was median 57 (iqr 49–66). The median RTS (revised trauma score) on admission was 3.0 (iqr 0.0–7.0), with 23 (64%) presenting with SOL on admission. The probability of survival (P_s_) was estimated at a median of 6.4% (iqr 1.1–29.5%) for all patients.

The overall relationship between age of patients and their corresponding injury profile demonstrated a shattered distribution with no clear-cut pattern or correlation (Supplementary Fig. 1 ).

**Fig. 3:**
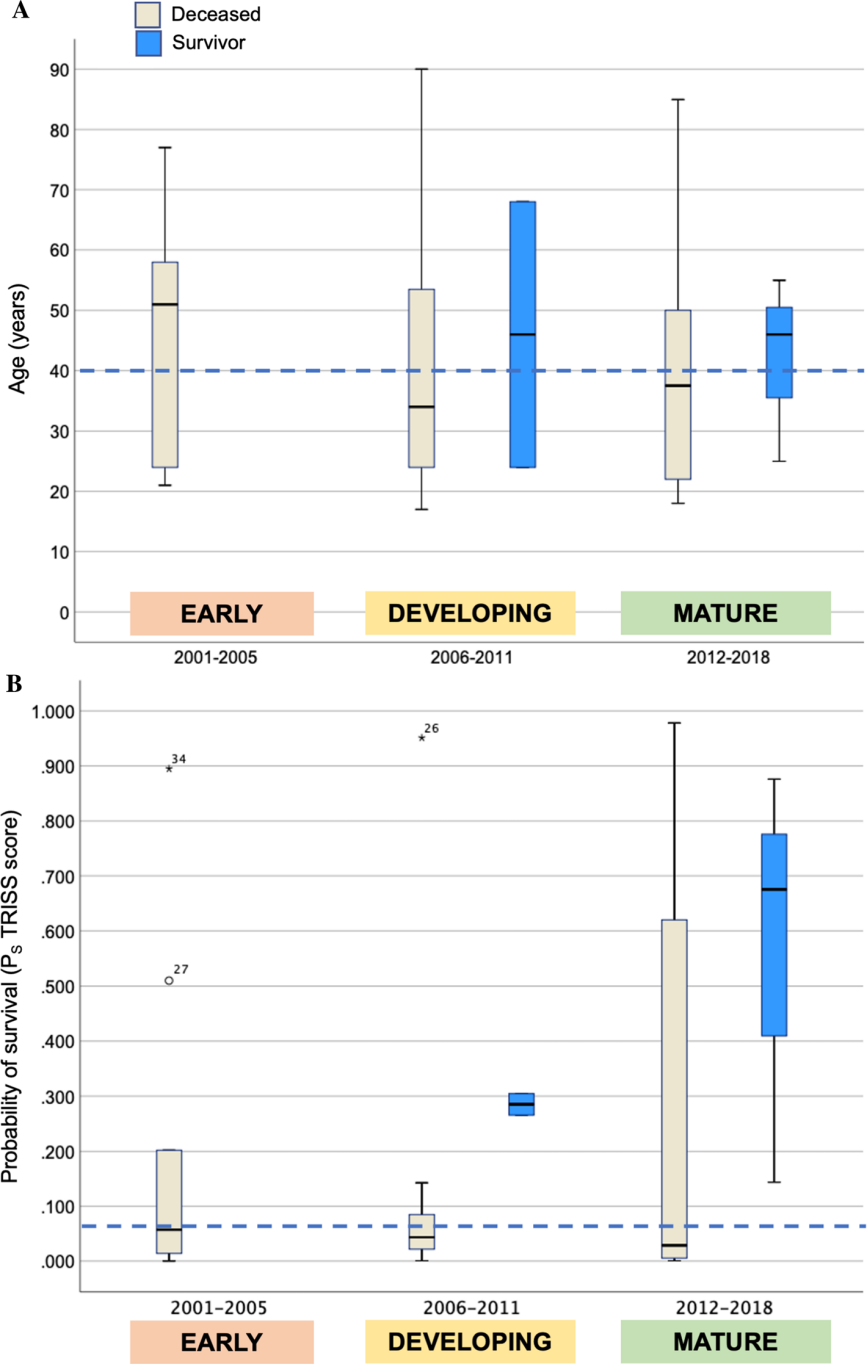
Age distribution and probability of Survival according to 3 time-dependent phases. *Legend* **a** The median age across the entire period was 40 years (blue, dotted line), with no significant difference between time-periods. In **b** is shown a non-significant increase in Ps over time, particularly for survivors. The median Ps value was very low (median Ps at 6.4%) for the entire cohort (blue, dotted line) indicating a critically ill and severely injured population

The patient characteristics and injury data for each time period are presented in Table 2. As shown, the number of survivors increased (Fig. 2) with each time period, as did the rate who had SOL on admission (from 40 to 77%) while the number of ‘elderly’ >55 years subject to an emergency thoracotomy decreased (Table 2). Most other characteristics remained unchanged throughout the period (Table 2, Fig. 3). In Fig. 2, some of the time-critical events in trauma education and training for developing and maturing of the trauma system and hospital preparedness are presented.

**Table 2:** Patient and procedure characteristics during 3 phases of system development

Characteristics	Early	Developing	Mature	*P*_*TREND*_^a^
**Period**	2001–2005	2006–2011	2012–2018	*n.a*
Years (*n*)	5	6	7	
**Procedures**^**b**^**,** *n*	*10*	*13*	*13*	0.279
Survivors	*0*	*2*	*3*	
**Gender**	3:7	4:9	2:11	0.605
Female:Male				
**Age** (median, iqr)	51 (24–59)	34 (23–65)	45 (24–51)	0.077
> 55 years, n (%)	5 (50%)	5 (31%)	1 (7.7%)	
**Mechanism**				
Blunt	7	11	10	0.702
Penetrating	3	2	3	
**Injury severity**	35 (26–52)	50 (37–59)	35 (23–62)	0.207
ISS (median, iqr)				
NISS (median, iqr)	57 (46–68)	66 (50–66)	50 (34–62)	0.255
**RTS, admission**	0.0 (0.0–4.8)	3.0 (0.5–8.0)	6.0 (0.0–8.0)	0.801
Median, iqr				
**LOMI** (*n*) Thoracic	4	10	9	0.166
Any other	6	3	4	
**SOL on admission** Present, *n* (%)	4/10 (40%)	9/13 (69%)	10/13 (77%)	0.166
**TRISS (mean)**	**19.1**	**15.7**	**31.4**	**0.422**
**P**_**S**_ in % (median, iqr)	4.4 (0.5–27.9)	6.8 (0.2–20.4)	8.4 (0.8–65.4)	0.877

Data are presented as median (interquartile range, IQR) if not otherwise stated

^a^P_trend_ indicating differences between groups investigated as a trend between time periods

^b^Resuscitative emergency thoracotomy; either as anterolateral thoracotomy, sternotomy or clamshell

ISS denotes injury severity score; NISS denotes New injury severity score; Ps (denotes probability of survival from the Trauma revised injury severity score; TRISS); RTS denotes revised trauma score; LOMI denotes ‘Location Of Major Injury’; SOL denotes “signs of life” on admission

## Discussion

In this study, we present structural and temporal changes to trauma team training consisting of team simulation and procedure training developed through 3 phases of trauma system development and maturation. We demonstrate improved outcome for one time-critical emergency procedure done at low frequency during this time period, associated with concurrent changes in trauma team and individual skills training. While this association is not intended to suggest a direct causality link, we believe it supports the role of structured training and simulation in trauma care by the associated outcomes.

Indications have slightly changed over time, indicated by fewer elderly patients and fewer patients with no SOL on admission subject to emergency thoracotomy. This should be viewed as a wanted effect of the focused training and education. Clearly, the elderly have less physiological reserve and for any emergency surgery and trauma intervention one should carefully consider the balanced risk–benefit to avoid futility [35]. One US study found no survivors in patients >57 years of age who underwent emergency thoracotomy [36]. Of note, the two oldest survivors in the current series were 55 and 68 years, respectively. We had no survivors in any of the very elderly patients (77, 85 and 90 years) having emergency thoracotomies performed, although 2 of 3 had SOL on admission and thoracic injury as LOMI. These patients had high ISS and NISS score, and all had sustained blunt injury mechanisms. We conclude that emergency thoracotomy would best be withheld in similar cases in the future. We also concur with the proposed guidelines [37] that when (a) prehospital CPR exceeding 10 min after blunt trauma without a response, (b) prehospital CPR exceeding 15 min after penetrating trauma without a response, and (c) when asystole is the presenting rhythm, and there is no pericardial tamponade there is no indication of resuscitative emergency thoracotomy as the outcome is considered futile.

The low frequency at which this procedure is performed is one of the barriers to both practice evaluation and outcome assessment. However, the rarity of the procedure may reflect the real-life situation in many geographical regions, even in level I centers. In rural and less densely populated regions this event may be rare, but may still produce favorable outcomes, such as reported in a study from Iceland [38]. In Germany, almost 50% of all emergency thoracotomies were done outside the supra-regional trauma centers [14], with one in ten done in a local hospital. Moreover, in a recent study from the Pennsylvania trauma system covering 28 hospitals, some 10 centers did on average <2 procedures/year (3 of which were designated level I centers) and only 3 centers consistently did >10 procedures per year [14]. Consequently, the rarity of this procedure and the urgency of its nature do not permit a ‘centralization’ as a remedy to increase the chance of success for critically injured patients. We believe that training and simulation are key to enhance performance.

Stavanger University Hospital has had a long-standing focus on the trauma chain of survival[39] from prehospital to rehabilitation, with several faculties involved in core trauma topics ranging from prehospital care [40–42] and resuscitation [43, 44] to injury management. Compared to the US and other mature systems, trauma systems in Scandinavia may have only matured more recently [45], and the study period has seen several structural changes in Norway, including commencement of a national trauma plan (first version in 2006, revised in 2016) with designated centers for trauma care yet a somewhat slow adoption. While implementation has been documented to be slow among local hospitals [46], no similar quality assessment has been done for all of the trauma centers.

Demonstrating a real effect of the efforts put into training and education is difficult and near impossible in terms of return on saved lives and limbs. Hence the discussion of ATLS-principles [29, 47–49], teaching practices and the impact beyond confidence boosting for the trainee. Still, we practice because we believe in return on investment. Continued training also makes sense in the light of increased focus on competence-based practice derived from deliberate training [50]. While hard to prove, we believe the current study may point to return on investment due to an increased and steadfast focus on systems improvement year-on-year in our hospital. We believe this practice to be transferrable to other hospitals and other settings, independent of geography and population density.

Some limitations of the study have to be addressed. One is the small number of patients over a long time. However, this would be the case for the majority of hospitals that receive injured patients. Measuring effect of structured training to such a rare procedure can hardly be done through trials of any sort. Of note, we prospectively collected data since 2004 in the trauma registry. Further, due to the low number of events, statistical power does not allow to draw any causality beyond associations. Nonetheless, we have found associations with the time periods and maturing of the trauma center, however, we do not claim any direct causality. Despite the low volume, we believe the data can be used by several other hospitals to encourage systematic and structured trauma training. Also, even some trauma centers in the US have comparable low volumes of these procedures, so incentives to practice and improve should be present across all levels and domains.

## Conclusion

Increased focus on training and simulation and maturation of the trauma system was associated with improved outcome for a rare, but potentially life-saving intervention. While causality cannot be claimed, the findings may be transferrable to similar settings and may encourage training and deliberate practice to enhance performance.

## Supplementary Information

Age-dependent distribution of injury scores for survivors and non-survivors.*Legend* Shown are the distribution in scatterplots of (A) Revised Trauma Score (RTS) , (B) the Injury Severity Score (ISS); (C) the Probability of Survival (Ps) and (D) scatterplot of NISS to age with lines representing the quadratic relations. In the latter, there is a decline in NISS with increasing age, while no such relationship is found for survivors. The interpretation is hampered by low numbers yet serves to show distribution among variables related to outcome. (TIFF 6574 kb)
