# Supplementary figures and images for: Structured and Systematic Team and Procedure Training in Severe Trauma: Going from ‘Zero to Hero’ for a Time-Critical, Low-Volume Emergency Procedure Over Three Time Periods
Source: World J Surg. 2021 Feb 10;45(5):1340–8. doi: 10.1007/s00268-021-05980-1 (PMC8026408; doi:10.1007/s00268-021-05980-1)

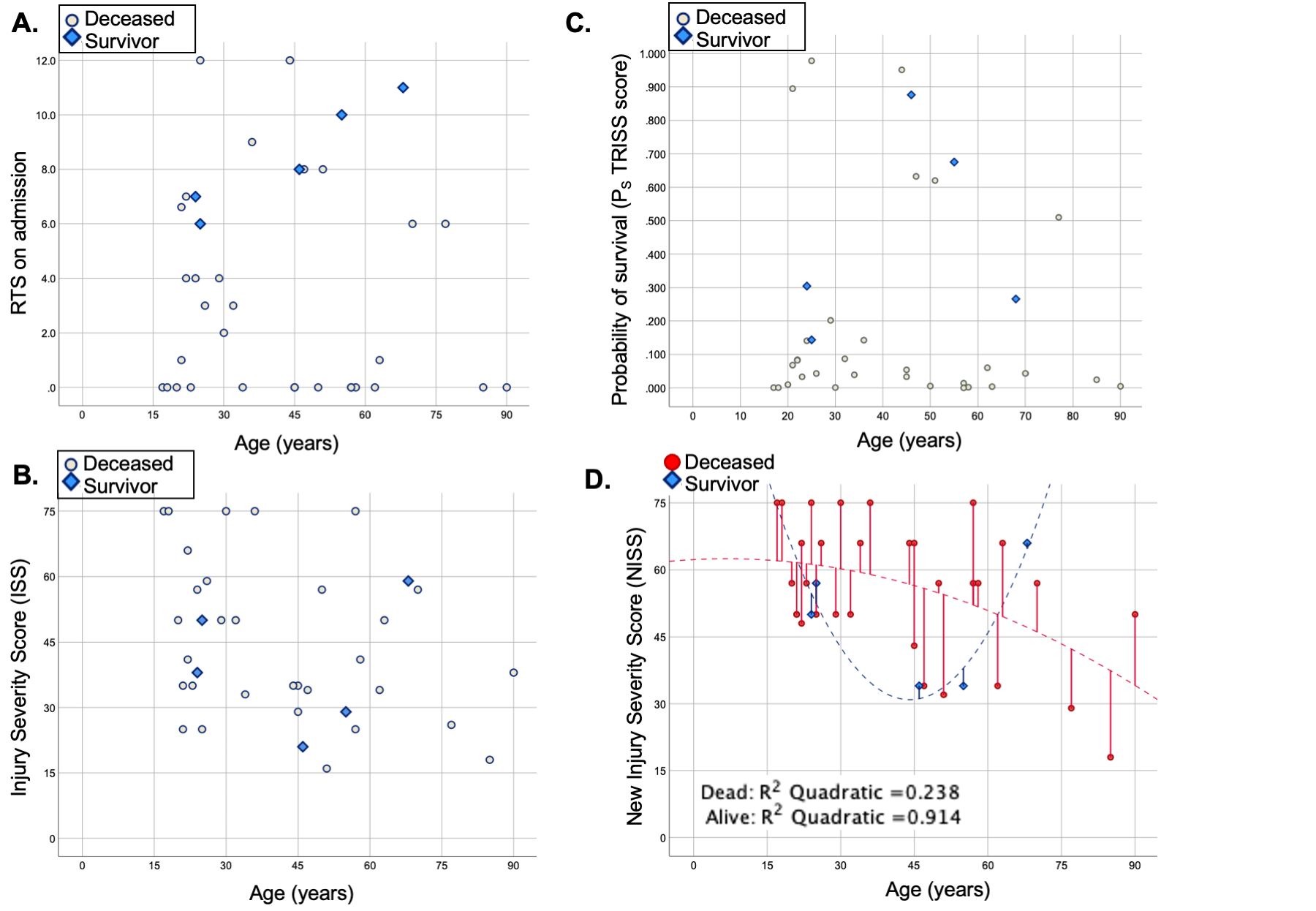

Supplement: Supplementary file 1 — Age-dependent distribution of injury scores for survivors and non-survivors.Legend Shown are the distribution in scatterplots of (A) Revised Trauma Score (RTS) , (B) the Injury Severity Score (ISS); (C) the Probability of Survival (Ps) and (D) scatterplot of NISS to age with lines representing the quadratic relations. In the latter, there is a decline in NISS with increasing age, while no such relationship is found for survivors. The interpretation is hampered by low numbers yet serves to show distribution among variables related to outcome. (TIFF 6574 kb) [file 268_2021_5980_MOESM1_ESM.tiff]
